# Higher Adalimumab Trough Levels Are Associated with Histologic Remission and Mucosal Healing in Inflammatory Bowel Disease

**DOI:** 10.3390/jcm12216796

**Published:** 2023-10-27

**Authors:** Rochelle Wong, Lihui Qin, Yushan Pan, Prerna Mahtani, Randy Longman, Dana Lukin, Ellen Scherl, Robert Battat

**Affiliations:** 1 Division of Gastroenterology, Department of Medicine, Weill Cornell Medical College, New York, NY 10065, USA; 2 Department of Pathology, Weill Cornell Medical College, New York, NY 10065, USA; 3 Division of Gastroenterology, Centre Hospitalier de l’Universite de Montreal, Montreal, QC H2X 0C1, Canada

**Keywords:** adalimumab maintenance, therapeutic drug monitoring, mucosal healing

## Abstract

(1) Many patients with inflammatory bowel disease (IBD) in endoscopic remission have persistent histologic activity, which is associated with worse outcomes. There are limited data on the association between adalimumab drug concentrations and histologic outcomes using validated histologic indices. We aimed to assess the relationship between adalimumab concentrations and the Robarts Histopathology Index (RHI). (2) Patients from a tertiary IBD center from 2013 to 2020 with serum adalimumab (ADA) trough concentrations measured during maintenance therapy (≥14 weeks) and a colonoscopy or flexible sigmoidoscopy with biopsies performed within 90 days of drug level were included. Blinded histologic scoring using the RHI was performed. Primary analysis assessed the relationship between adalimumab drug concentrations and histologic remission using receiver operating characteristic curve analysis. (3) In 36 patients (26 Crohn’s Disease, 9 ulcerative colitis, 1 indeterminate), median adalimumab concentrations were higher (17.3 ug/mL, 12.2–24.0) in patients with histologic remission compared to those without (10.3 ug/mL, 6.8–13.9, p = 0.008). The optimal ADA concentration identified using the Youden threshold was ≥16.3 ug/mL (sensitivity 70%, specificity 90%). Patients with ADA ≥ 16.3 ug/mL had higher histologic remission rates (78%) compared to lower ADA concentrations (14%, *p*= 0.002), as well as higher mucosal healing rates (86%) compared to lower levels (12%, *p* = 0.001). Symptoms correlated weakly and non-significantly with both histologic (RHI) scores (r = 0.25, *p* = 0.2) and adalimumab concentrations (r = 0.05, *p* = 0.8). (4) The current study demonstrated that higher serum adalimumab concentrations (≥16.3 ug/mL) are needed for histologic remission and mucosal healing assessed using the RHI.

## 1. Introduction

Ulcerative colitis (UC) and Crohn’s disease (CD) are chronic inflammatory bowel diseases (IBD) [1,2]. Endoscopic healing in IBD has consistently been associated with reductions in corticosteroid use, hospitalization, and surgery. Thus, endoscopic healing is the recommended primary treatment target for IBD [3,4,5,6,7]. Additionally, improved long-term outcomes are associated with more stringent endoscopic outcomes with a complete absence of disease activity [8,9,10,11,12,13]. Despite this fact, significant proportions of patients with IBD in endoscopic remission have persistent histologic activity, which is associated with higher rates of symptomatic relapse, corticosteroid use, surgery, and dysplasia [14,15]. Thus, incorporating histology into management is now recommended, and regulatory authorities require the term “mucosal healing” to refer to achieving both endoscopic and histologic remission [16]. Consequently, there has been significant interest in the use of validated histology instruments, such as the Robarts Histopathology Index (RHI), to assess histologic remission [17].

Therapeutic drug monitoring (TDM) has been demonstrated to optimize therapies to maintain efficacy in IBD, in which there are limited existing therapies [18]. Clinical situations during which TDM can be helpful include treatment failure, after successful induction and transition into maintenance therapy, assessing timing for a drug holiday, or during clinical remission when subsequent activity results would change management. Tumor necrosis factor (TNF) antagonist trough and anti-drug antibody concentrations are used in TDM and have been associated with important outcomes in IBD [19]. There are various strategies for providers to utilize TDM that are currently being studied. The standard of care currently involves empiric dose escalation of anti-TNF therapy if the patient does not achieve a response. Reactive TDM, where providers use drug concentration levels and antidrug antibodies to guide decision-making, has been helpful for patients who are suspected or confirmed to have a loss of response to therapy [20]. In contrast, proactive drug monitoring, where the drug is titrated to a target concentration, has been associated with better clinical outcomes, reduced risk of treatment failure, and lower risk of developing antidrug antibodies [21,22].

In both Crohn’s disease (CD) and ulcerative colitis (UC), TNF antagonists, such as adalimumab (ADA), are often required to induce and maintain remission. Adalimumab has been found to be effective in achieving and maintaining clinical remission for both CD [23] and UC [24,25] patients, including those who have been treated with prior anti-TNF therapy. Various studies have been published on the optimal therapeutic drug level for adalimumab to achieve clinical, endoscopic, and histologic remission. Levels of 4.8 ug/mL have been associated with clinical remission and >7.5 ug/mL for endoscopic remission [26]. For histologic remission, one study found drug levels >7.8 ug/mL were associated with histologic healing, using standard-of-care pathologist assessment for the absence of microscopic inflammatory infiltrate to define histologic remission but no formal histologic scoring criteria [27]. This initial study suggests higher concentrations may be needed to achieve deeper levels of remission. Another study showed that adalimumab drug concentrations >13.9 ug/mL at week 4 were associated with serological remission at week 24, consistent with emerging literature suggesting that higher concentrations of anti-TNF therapy may be needed to achieve a response [28,29].

However, despite the success of adalimumab therapy to induce and maintain remission, significant proportions of patients experience either primary non-response or secondary loss of response to anti-TNF therapy [30]. There are limited exposure-response data on adalimumab for validated histologic endpoints [31,32]. A recent randomized controlled trial found reduced efficacy of adalimumab relative to vedolizumab to achieve histologic remission defined using the RHI [33]. A potential explanation for suboptimal histologic outcomes with adalimumab may be inadequate drug concentrations. However, data on the relationship between serum adalimumab concentrations and histologic outcomes with validated indices are lacking. The RHI is a responsive indicator of histologic disease and treatment response in UC and CD, [33,34,35] with similar test characteristics to other histologic indices [36] and validated against endoscopy [37,38]. The RHI has been deemed appropriate to measure histological disease activity in CD [39] and utilized in landmark CD trials.

This study aimed to assess the relationship between serum drug concentrations of adalimumab and a validated histologic disease activity index in patients with IBD using prospectively collected, blinded and objective histologic scores.

## 2. Materials and Methods

### 2.1. Study Population

In this retrospective study, patients from a tertiary IBD center from 2013 to 2020 with adalimumab (ADA) trough drug concentrations measured during maintenance therapy (≥14 weeks) and a colonoscopy or flexible sigmoidoscopy with biopsies performed within 90 days of drug level were included. A chart review was performed for demographic data, medication and surgical history, and disease characteristics.

### 2.2. Data and Outcome Definitions

Serum adalimumab trough concentrations were measured using a homogenous mobility shift assay (Prometheus Laboratories, San Diego, CA, USA). Drug levels were drawn during maintenance therapy for routine drug monitoring, regardless of clinical symptoms or clinical remission. Additional bloodwork was drawn to evaluate for active inflammation if the patient was symptomatically active.

For inclusion criteria, trough levels were defined as drug concentration levels drawn within 7 days prior to the next administration of ADA for patients receiving therapy every 2 weeks, or on the day prior to the next administration for those on weekly injections. However, because the standard practice at our center is to collect serum adalimumab concentrations within 1 day prior to drug administration, the median time of drug concentration measurement prior to the next dose reflected a more stringent trough definition (1.5 days) in this study.

For patients with colonoscopy or flexible sigmoidoscopy performed within 90 days of drug level, histologic scoring using the RHI was performed by a blinded pathologist on the biopsies obtained during ileo-colonoscopy [15,17,34]. Biopsies for CD were taken from endoscopically inflamed segments, or at random if no endoscopic inflammation existed, from at least one segment throughout the ileum and/or colon. Biopsies for UC were also taken from endoscopically inflamed segments, or at random if no endoscopic inflammation existed, from the colon with at least one biopsy from the rectum, given the continuous pattern of inflammation from the rectum in this disease. Additional biopsies were taken from areas that appeared most endoscopically active or affected, such as the presence of ulcers or erythema, in order to accurately assess for inflammation.

Rates of endoscopic remission, defined as the absence of ulcers for CD [3,16] and a Mayo endoscopic score of 0 for UC [3,16] were assessed. Histologic remission, defined as RHI = 0, was also assessed [17,40]. Mucosal healing (MH) was defined as achieving both endoscopic and histologic remission. Rates of clinical (symptomatic) remission were assessed, as defined using a Harvey Bradshaw Index of 4 or less for patients with CD or a partial Mayo score of 2 or less for patients with UC.

### 2.3. Statistical Analysis

Primary analysis assessed the diagnostic accuracy of adalimumab drug concentrations for histologic remission using receiver operating characteristic curve analysis. Outcome proportions were compared above and below identified optimal (Youden) thresholds using Fisher’s exact test. Rates of endoscopic remission and mucosal healing (achieving both endoscopic and histologic remission) were additionally compared using the identified threshold. A *p*-value < 0.05 was considered significant.

All statistical analyses were performed using STATA SE 15.1 (Statacorp, College Station, TX, USA).

### 2.4. Ethics

All authors had access to the study data and reviewed and approved the final manuscript. Study protocol and materials were approved by the institutional review board at Weill Cornell Medicine. The study was conducted in accordance with the Declaration of Helsinki and approved by the Institutional Review Board of Weill Cornell Medicine (Protocol code 20-04021893 and date of approval 5 August 2020). All patients provided written informed consent.

## 3. Results

Thirty-six patients were included (26 CD, 9 UC, 1 indeterminate, Table 1). The median cohort age was 34 years old, and 56% of patients were female. The median ADA drug concentration was 11.1 ug/mL (IQR: 7.0–15.5 ug/mL). The median time from treatment initiation to drug concentration measurement was 103 weeks (IQR 25–75 = 35.6–286). The median time of drug concentration measurement prior to the next dose was 1.5 days. Endoscopic remission was noted in 7/24 (29%) of CD patients and 1/4 (25%) of UC patients. The median RHI score was 8.5 (IQR 25–75 = 0–21.8) and histologic remission was achieved in 10/30 (33%) of patients. Of the 24 patients with both endoscopic and histologic data available, 8 patients (33%) achieved mucosal healing (endo-histologic remission). Median adalimumab concentrations were 12.1 ug/mL in patients with symptomatic remission, 13.9 ug/mL in patients with endoscopic remission, 17.3 ug/mL in patients with histologic remission, and 19.6 ug/mL in patients with complete mucosal healing (endo-histologic remission).

### 3.1. Relationship between Adalimumab Concentrations and Histology

**Figure 1:**
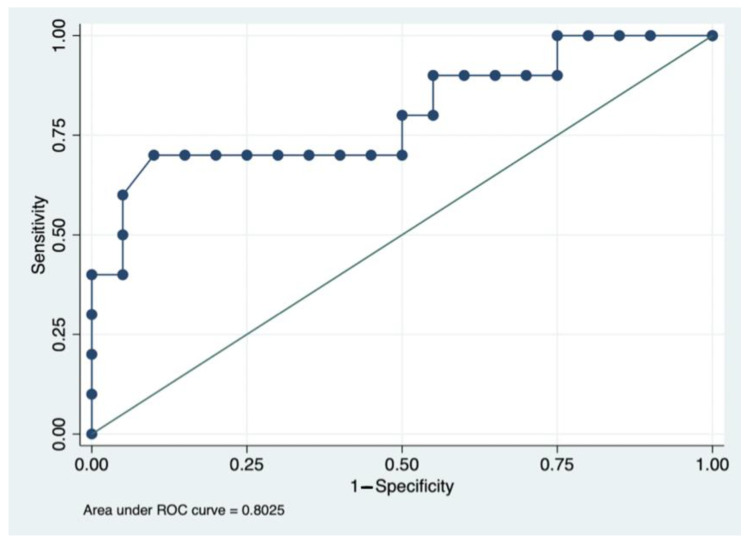
Area Under the Receiver Operator Curve Analysis for Histologic Remission (RHI = 0) and adalimumab drug concentrations.

**Figure 2:**
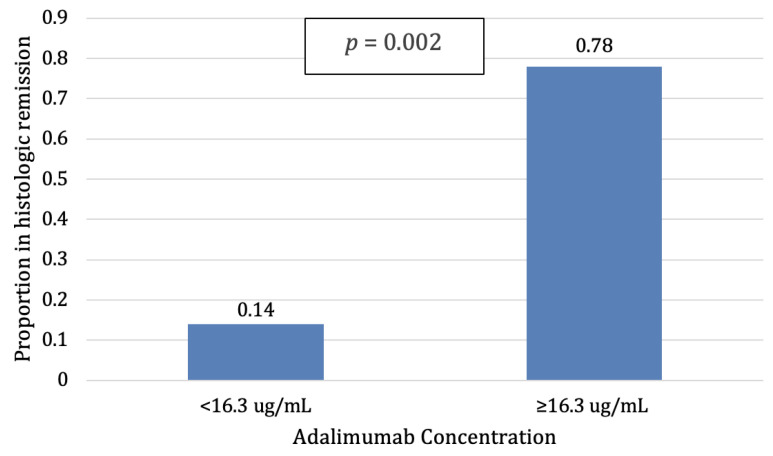
Patients with higher adalimumab (ADA) concentrations achieved statistically significantly higher histologic remission rates than patients with lower ADA concentrations (*p* = 0.002).

Median adalimumab concentrations were higher (17.3 ug/mL, 12.2–24.0) in patients with histologic remission compared to patients without histologic remission (10.3 ug/mL, 6.8–13.9, *p* = 0.008). The area under the curve for ADA concentrations to identify histologic remission was 0.80 (95% CI 0.61–0.99, Figure 1). The optimal ADA concentration identified using the Youden threshold was ≥16.3 ug/mL (sensitivity 70%, specificity 90%, positive likelihood ratio 7.0, negative likelihood ratio 0.33). Patients with ADA ≥ 16.3 ug/mL had higher histologic remission rates (78%) compared to patients with lower ADA concentrations (14%, *p*= 0.002, Figure 2).

In quartile analysis of drug concentrations associated with the primary outcome, 17% (1/6) of patients achieved mucosal healing in quartile 1 (0–7 ug/mL), 17% (1/6) of patients achieved mucosal healing in quartile 2 (7–12.3 ug/mL), 14% (1/7) of patients achieved mucosal healing in quartile 3 (12.4–16.3 ug/mL), and 100% (5/5) achieved mucosal healing in quartile 4 (16.4–26.4 ug/mL).

### 3.2. Relationship between Adalimumab Concentrations and Endo-Histologic Outcomes

**Figure 3:**
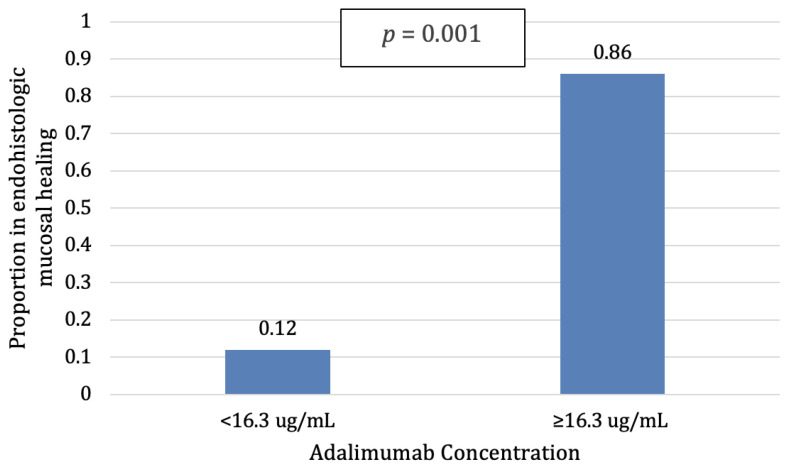
Patients with higher ADA concentrations achieved statistically significantly higher mucosal healing (endohistologic remission) rates than patients with lower ADA concentrations (*p* = 0.001).

The median adalimumab concentrations were significantly higher (19.6 ug/mL, 14.6–24.9) in patients with complete mucosal healing (both endoscopic and histologic remission) compared to patients without complete mucosal healing (10.3 ug/mL, 5.9–13.9, *p* = 0.009). Using the previously identified threshold, patients with an adalimumab concentration ≥16.3 ug/mL also had higher rates of complete mucosal healing (86%) compared to patients with lower adalimumab concentrations (12%, *p* = 0.001, Figure 3).

Using the previously identified threshold, patients with an adalimumab concentration ≥16.3 ug/mL had higher endoscopic remission (100%) compared to patients with lower adalimumab concentrations (57%, *p* = 0.04). In addition, the median adalimumab concentrations were numerically higher (13.9 ug/mL, 7.7–17.0) in patients with endoscopic remission compared to patients without endoscopic remission (9.1 ug/mL, 6.1–13.0, *p* = 0.16).

### 3.3. Relationship between Adalimumab Concentrations and Symptomatic Outcomes

The median adalimumab concentrations were similar between patients with (12.1 ug/mL, 5.9–14.5) and without (10.9 ug/mL, 8.9–16.0) symptomatic (clinical) remission. The area under the curve for ADA concentrations to identify symptomatic remission was 0.45 (95% CI 0.24–0.66). Symptoms correlated weakly and non-significantly with both histologic (RHI) scores (r = 0.25, *p* = 0.2) and adalimumab concentrations (r = 0.05, *p* = 0.8).

### 3.4. Relationship between Adalimumab Concentrations and Composite Outcome of Mucosal Healing and Clinical Remission

**Figure 4:**
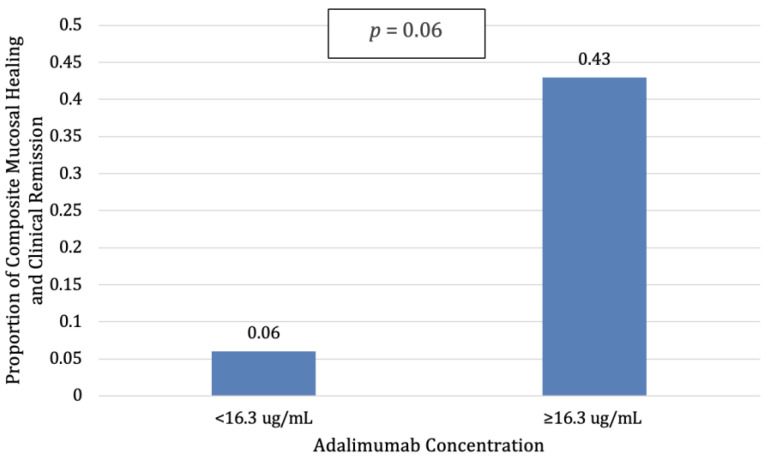
Patients with higher ADA concentrations trended towards improved mucosal healing and clinical remission when compared to patients with lower ADA concentrations (*p* = 0.06).

The median adalimumab concentrations for patients with both mucosal healing and clinical remission was 18.9 ug/mL, IQR 13.7–22.7, while the median adalimumab concentration for patients without both was 11.2 ug/mL, IQR 7–14.8, *p* = 0.15. Similar numerical differences existed with a smaller sample size of those with endoscopic, symptomatic, and histologic data. Using the previously identified threshold, patients with an adalimumab concentration ≥16.3 ug/mL trended toward higher mucosal healing and clinical remission (43%) compared to patients with lower adalimumab concentrations (6%, *p* = 0.06, Figure 4).

## 4. Discussion

Histologic remission and MH may better predict relapse and long-term outcomes than clinical or endoscopic remission alone [19,30]. Thus, histopathology has been suggested as an adjunctive goal in therapeutic targets in management guidelines [3]. Consequently, understanding the exposure-response relationship between common biologic therapies and these outcomes is important. Adalimumab has been shown to have inferior histologic outcomes to other agents [33]. However, data on the relationship between validated histologic disease activity indices and adalimumab drug concentrations are lacking. The current study was the first to uniquely describe and demonstrate a significant relationship between adalimumab maintenance trough concentrations and histologic outcomes using a validated histologic index.

Therapeutic drug monitoring (TDM), defined as using serum drug concentrations and the presence of anti-drug antibodies to guide management, can be helpful in patients with both a primary non-response or secondary loss of response to biologic therapy [41]. As TDM becomes more incorporated into clinical practice and management, it will be important to clarify the target goal for patients to achieve histologic remission and mucosal healing. The current recommended target adalimumab concentration is 7.5 ug/mL to achieve endoscopic remission [42]. However, this level is best correlated with the lack of endoscopic lesions and may not achieve mucosal healing (endohistologic remission) due to low sensitivity [27]. Our study suggests that a higher than traditional serum ADA target (≥16.3 ug/mL) is needed to achieve histologic remission. Several prior studies have reported on the higher maintenance of adalimumab concentrations, which achieved higher rates of histologic remission and mucosal healing in IBD patients [27,43]. However, the main strength of our study is that it is the first to use a validated histologic scoring tool, as well as a blinded histologic disease activity assessment, in contrast to previous studies that lacked validated histologic scoring tools and utilized retrospectively reviewed pathology [27].

One strength of our study was the use of stringent endoscopic and histologic outcomes. It has been suggested that early proactive monitoring of mucosal inflammation and mucosal healing within 6 months of biologic initiation is associated with a reduction in complications at 24 months, including corticosteroid use, change in biologic, IBD-related hospitalization, or surgery [44]. However, rather than using noninvasive monitoring, such as fecal calprotectin, endoscopic evaluation, or cross-sectional radiographic enterogarphy, our primary outcome was the most stringent of histologic remission, defined as RHI of 0. This has been already strongly associated with patient clinical and endoscopic remission status [40]. An RHI score of 0 ensures complete histologic remission outcomes. Our use of mucosal healing, defined as endo-histologic remission, as an endpoint also better reflects current practice. Although not formally defined as a therapeutic target, histopathology showing active mucosal inflammation on biopsy may increase clinical suspicion for underappreciated endoscopic disease activity, and prompt treatment adjustments or earlier disease activity reassessments.

Limitations of the current study include its retrospective study design, its limited sample size, and a low proportion of patients being in complete endo-histologic remission. However, between-group differences in histologic and MH rates were not only statistically significant but also had strikingly large numerical differences. It is important to note the evolving definitions of mucosal healing [45]. We define mucosal healing in our study to be combined endoscopic and histologic remission, in line with recent Food and Drug Administration (FDA) recommendations. Prior studies have used similar terminology to define only endoscopic remission [46,47]. Our study also defines endoscopic remission for UC as Mayo 0. Mayo 0 shows a lower risk of clinical relapse than Mayo 1, but no differences in risk of hospitalization or IBD surgery [48]. Drug concentration thresholds may differ depending on the outcome of interest. Ungar et al. defined mucosal healing as endoscopic score remission and found that ADA serum levels > 7.1 ug/mL predicted endoscopic MH with 85% specificity, while the current study data suggest a higher ADA level is required to achieve both endoscopic and histologic remission.

Another limitation to consider is that a serum ADA target of 16.3 ug/mL may be difficult to achieve. Proactive therapeutic drug monitoring, which utilizes dose escalation to achieve a threshold concentration regardless of disease activity, may be a strategy to achieve higher adalimumab concentrations appropriately and cost-effectively [49,50]. Testing has more commonly been performed at trough, as the presence of the drug can interfere with the detection of anti-TNF antibodies. The timing of when to measure drug serum concentrations can also be unclear when practicing therapeutic drug monitoring. However, recent data suggest that serum adalimumab concentrations are stable in the first 9 days after injection and can reasonably predict therapeutic trough drug levels, potentially allowing for earlier decision-making based on non-trough adalimumab levels [51,52]. One study by Kato et al. found serum ADA levels are predictive of clinical outcomes regardless of trough timing [53]. This may be helpful for patients on more frequent dosing of adalimumab, while the timing of drug levels may be more important to make for patients requiring longer follow-up. Future studies are needed to investigate the feasibility of this TDM practice.

## 5. Conclusions

To conclude, this study reports a serum ADA concentration that is higher than traditional targets (≥16.3 ug/mL) is associated with higher rates of histologic remission and mucosal healing.

## Figures and Tables

**Table 1:** Patient Cohort Demographics (*n* = 36).

**Demographics**	***n*** **(%)**
Median Age at Drug Level (years)	34
Gender (female)	20 (0.56)
**Type of IBD**	
Crohn’s Disease	26 (0.72)
Ulcerative Colitis	9 (0.25)
Indeterminate Colitis	1 (0.02)
**Adalimumab**	
Median Drug Level Concentration (IQR 25–75)	11.1 (7.0–15.5)
Median Dose (mg)	40
Median Frequency (every X weeks)	2
Median Days of Therapy (d)	718
Median Weeks of Therapy (wk)	103
**Age at Diagnosis**	
Age < or = 16	11 (0.31)
Age 17–40	15 (0.42)
Age > or = 41	8 (0.22)
Unknown	2 (0.06)
**Montreal Classification**	
**Crohn’s Disease (*n* = 26)**	
B1—inflamed, non-stricturing, non-penetrating	13 (0.50)
B2—stricturing	6 (0.23)
B3—fistulizing (penetrating)	7 (0.26)
CD: L1 ileal	5 (0.19)
CD: L2 colonic	3 (0.12)
CD: L3 ileocolonic	17 (0.65)
CD: L4 isolated upper GI disease	6 (0.23)
**Ulcerative Colitis (*n* = 9)**	
UC: left-sided (rectum to splenic flexure)	5 (0.56)
UC: Extensive (beyond splenic flexure, including ascending/transverse colon)	4 (0.44)
**Endoscopy**	
CD: Presence of ulcers (lack of remission)	7/24 (0.29)
UC: Mayo Score <2 (presence of remission)	1/4 (0.25)
**Histology**	
RHI score = 0 (histologic remission)	10/30 (0.33)
Median RHI Score	8.5
**Mucosal Healing**	
Endohistologic Remission	8/24 (0.33)
**Medication History**	
Previously used mesalamine	28 (0.78)
Previously used sulfasalazine	5 (0.14)
Previously used budesonide	14 (0.39)
Previously used 6-mercaptopurine	14 (0.39)
Previously used methotrexate	5 (0.14)
Previously used azathioprine	6 (0.17)
Prior TNF exposure	13 (0.36)
Prior Vedolizumab exposure	1 (0.03)
Prior steroid (prednisone) use	19 (0.53)
**Surgical History**	
Previous IBD-related abdominal surgery	12 (0.33)

## Data Availability

Data not publicly available. Data available on request.
